# Natural Biomolecules and Protein Aggregation: Emerging Strategies against Amyloidogenesis

**DOI:** 10.3390/ijms131217121

**Published:** 2012-12-14

**Authors:** Antonella Sgarbossa

**Affiliations:** Institute of Biophysics, CNR, Italian National Research Council, Via G. Moruzzi 1, 56124 Pisa, Italy; E-Mail: antonella.sgarbossa@pi.ibf.cnr.it; Tel.: +39-0506213021; Fax: +39-0503152760

**Keywords:** proteins, peptides, self-assembly, misfolding, aggregation, amyloid, fibrillogenesis, natural molecules, polyphenols, aromatic molecules

## Abstract

Biomolecular self-assembly is a fundamental process in all organisms. As primary components of the life molecular machinery, proteins have a vast array of resources available to them for self-assembly in a functional structure. Protein self-assembly, however, can also occur in an aberrant way, giving rise to non-native aggregated structures responsible for severe, progressive human diseases that have a serious social impact. Different neurodegenerative disorders, like Huntington’s, Alzheimer’s, and spongiform encephalopathy diseases, have in common the presence of insoluble protein aggregates, generally termed “amyloid,” that share several physicochemical features: a fibrillar morphology, a predominantly beta-sheet secondary structure, birefringence upon staining with the dye Congo red, insolubility in common solvents and detergents, and protease resistance. Conformational constrains, hydrophobic and stacking interactions can play a key role in the fibrillogenesis process and protein–protein and peptide–peptide interactions—resulting in self-assembly phenomena of peptides yielding fibrils—that can be modulated and influenced by natural biomolecules. Small organic molecules, which possess both hydrophilic and hydrophobic moieties able to bind to peptide/protein molecules through hydrogen bonds and hydrophobic and aromatic interactions, are potential candidates against amyloidogenesis. In this review some significant case examples will be critically discussed.

## 1. Introduction

Several natural molecules derived from animals, plants, fungi, bacteria and other organisms show an extraordinary structural diversity, as well as a broad range of biological activities. They actually represent one of the major sources of therapeutic remedies and important tools for developing innovative drugs. In fact, natural compounds have been designed by evolution to have functional groups well-fitting biomolecular frameworks. Therefore, they can be considered privileged structures able to specifically interact with biological membranes and macromolecules, mostly proteins [1].

In recent years, phytochemicals and/or small natural molecules derived from dietary constituents have been extensively investigated and checked for their therapeutic use in a wide range of human diseases. A large number of studies on their biological activity, as well as on their efficacy to prevent, minimize, and possibly defeat several pathologies, have been carried out. Small phenolic compounds and naturally occurring antioxidants, for example, have been intensively studied for their beneficial as well as controversial effects in reducing the risk of different diseases, including cancer, diabetes, cardiovascular and neurological pathologies [2–5]. One of the major properties of the phenolic compounds is their ability to interact with peptides and proteins, thus modifying their structural properties and altering their biological activities. Protein misfolding and self-assembly in insoluble amyloid fibrillar structures are processes at the basis of many severe diseases such as Alzheimer’s, Parkinson’s, or Huntington’s disease, as well as diabetes and systemic amyloidosis [6]. Protein–protein and peptide–peptide interactions yielding amyloid fibrils can be modulated and influenced by small organic molecules that might also be effective tools in elaborating therapeutic strategies against pathological amyloidogenesis [7].

In this review, the importance of protein misfolding and aggregation in amyloid-related diseases will be dealt with, and some of the most recent findings on the effects of natural molecules on these processes will be discussed.

## 2. Protein Folding and Aggregation

### 2.1. Protein Folding

In biology, molecular self-assembly is a fundamental process which governs and drives the most important functions necessary for life in all the organisms. The formation of cell membranes and multi-protein complexes such as ribosomes or RNA polymerases, the folding of nucleic acids into DNA, as well as of polypeptide chains into proteins, are biological processes in which molecules spontaneously form ordered structures that are either thermodynamically stable or metastable [8].

Proteins represent the most abundant molecules in biological systems: 20 different amino acids can join together in different combinations and sequences to form a large diversity of proteins playing a vast set of biological functions. Because each amino acid can occur many times along the polypeptides sequence and there are no restrictions on the length of the chain, the number of the possible combinations for the formation of proteins is truly enormous. Natural proteins are only a small part of the immense amount of the possible polypeptide sequences. They are selected during evolution on the basis of their functional advantages. In fact, under physiological conditions, proteins are able to fold to a specific tightly packed three-dimensional structure possessing a broad range of functions and a high degree of selectivity. This biologically active structure is called “native”.

According to the Anfinsen’s thermodynamic hypothesis, the native structure of a protein is a thermodynamically stable structure; it depends only on the amino acid sequence and on the physico-chemical properties of the solution [9]. Hydrogen bonds, ion pairs, van der Waals forces, and water-mediated hydrophobic interactions between the side chains of the different amino acids along the polypeptide chains concur to determine the secondary structure which then encodes the tertiary structure. In the last 30 years, the idea has emerged that the hydrophobic interaction is the dominant driving force that plays a major role in the folding process [10,11]. The folding of proteins, in particular of the larger ones, is likely to initiate independently in different local regions or domains in a modular way. Then appropriate native-like interactions within and between the domains take place in a cooperative step to form the correct native packed structure where water is excluded from the protein core. In this perspective, the tertiary structure is not considered a consequence of the secondary one but almost a cause of it. As protein folding proceeds, partially folded or misfolded intermediates populate the pathway toward the native structure or kinetically stable, misfolded conformations that may require a substantial reorganization before reaching the native state [12,13].

In the highly crowded environment of the living cell the correct protein folding may be influenced by the presence of high local concentrations of other macromolecules and structures necessary for cell survival and replication. Inappropriately folded protein intermediates may aggregate with each other or associate with other cellular components, leading to the suppression of their biological functions and therefore, in most cases, to disease. In order to ensure and optimize the efficiency of folding, cellular chaperone machinery plays a key role in interacting, stabilizing or helping a non-native protein to acquire its native conformation [14,15]. Chaperone proteins look after and control the protein folding so that mistakes are minimized and defective proteins are degraded and removed by the ubiquitin-proteasome system [16,17]. Failure of these functions causes protein aggregation and may lead to cell pathology and disease.

### 2.2. Protein Aggregation

Misfolded proteins which escape the intracellular quality control system, tend to aggregate in insoluble clusters [18]. This is mainly due to the exposition of hydrophobic amino acid residues and regions mostly hidden in the native state. The association of two or more non-native peptide/protein molecules, largely driven by the hydrophobic interactions, gives rise to the formation of amorphous structures with a granular morphology as well as of highly ordered, fibrillar aggregates called amyloid with a predominantly β-sheet secondary structure in which β-strands run perpendicular to the long fibril axis (cross-β structure).

In the last 20 years, an increasing scientific interest in protein aggregation has prompted new insights into the physicochemical properties of protein folding. In fact, it is now well recognized that aggregation is not simply the direct consequence of an unfolding process. In many cases it is linked to a significant structural protein rearrangement with the loss of the native structure, *i.e.*, misfolding, and the adoption of a β-rich structure, *i.e.*, amyloid fibrils formation. On the other hand, there are a number of proteins that undergo amyloidogenesis through a “native-like” intermediate or that show extensive unstructured regions even when fully fibrilized. Furthermore, a number of severe and lethal diseases with a serious social impact are associated with misfolding and fibrillogenesis (Table 1) [19,20]. Among these, Alzheimer’s disease (AD) is the most known and it has an increasing incidence in the worldwide population. Also Parkinson’s disease (PD), as well as Huntington’s, prion diseases, amyloid lateral sclerosis, type II diabetes and systemic amyloidosis share the common hallmark of amyloid fibrillar aggregates.

Recent findings allow considering protein aggregation in a different perspective as it is not always noxious for living organisms. It is important to mention that functional amyloids have been also found in a wide range of organisms, from bacteria to mammals, with functions such as biofilm formation, development of aerial structures, scaffolding, regulation of melanin synthesis, epigenetic control of polyamines and information transfer [21].

## 3. Amyloid Fibrils and Disease

### 3.1. General Features

It is worthwhile emphasizing that amyloid fibrils, involved in different pathologies, are surprisingly similar in their morphology and structural properties, even though they originate from quite different proteins with different amino acids sequences, folded in very diverse and peculiar native structures [22]. They show birefringence upon binding several dyes as Congo red and Thioflavin T, often used in diagnosis. They appear as long unbranched fibers “whose repeating substructure consists of β-strands that run perpendicular to the fibre axis, forming a cross-β sheet of indefinite length” [23]. This structure was described in silk by Astbury more than 70 years ago [24] and, like silk, amyloid fibers of different proteins are extremely resilient to mechanical stress and not easily degraded by solvents and detergents. Recently, an ever-increasing number of studies indicate that most, if not all, peptides and proteins, even those not related to any disease, are able to misfold forming well-ordered amyloid aggregates [25,26].

As fibril formation can be reproduced *in vitro*, under appropriate conditions, without the assistance of cellular components, it represents another intriguing example of biomolecular self-assembly. Hence protein aggregation may be seen as the other side of the same coin, alternative to protein folding, where intermolecular rather than intramolecular interactions are privileged.

A recent study on the thermodynamic stability analysis of the amyloid forms of several peptides and proteins, indicate amyloid as the most stable structure, even under physiological conditions. In contrast to what has been hypothesized before, the protein native state may be only metastable with respect to amyloid formation, because of the high kinetic barriers associated with the *in viv*o self-assembly of polypeptide chains [27].

### 3.2. Amyloid Fibrillogenesis

**Figure 1:**
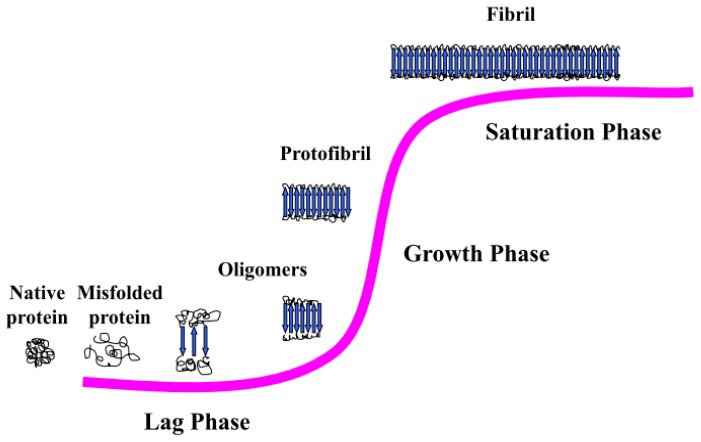
Schematic representation of the fibrillogenesis process.

Fibril formation is a polymerization process which can be simply described by a sigmoid curve, suggested to be a three-stage process consisting of protein misfolding, nucleation, and fibril elongation [28] (Figure 1).

The first phase is called “lag phase” during which the soluble species, usually monomers, associate to form nuclei and the transition from soluble native structure to oligomeric species with β-sheet conformation occurs.

The second phase is the “exponential phase” or “growth phase.” It consists of multiple steps through which the soluble species are progressively arranged at the ends of preformed β-sheet rich structures in a thermodynamically favorable process. In this phenomenon, a key role is played by forces common to all proteins, without any meaningful dependence on the specific peptide sequence: hydrophobic interactions, backbone hydrogen bonding, stacking interactions. Protofibrils are formed during the growth phase and represent the initial stable elements in the fibril formation pathway. There are two possible fibril growth mechanisms: β-sheet elongation, in which the fibril grows by adding individual peptides to the end of each β-sheet, and lateral addition, in which the fibril grows by adding an already-formed β-sheet to its side. Both mechanisms seem to play an equally significant initial role in fibril development. It has also been suggested that, consequently, two distinct phases in human fibrillogenesis can take place, where lateral growth of oligomers is followed by longitudinal growth into mature fibrils [29].

In the third phase, or “saturation phase,” the fibrils are completely formed and associate each other to form stable mature fibers [30].

The propensity of proteins and peptides to form amyloid fibrillar structures is correlated to their physicochemical properties such as net charge, secondary structure tendency, hydrophobicity and aromatic interactions [31]. The frequent occurrence of aromatic residues (tryptophan, tyrosine and phenylalanine) in different amyloid related polypeptides and the observation that short aromatic peptide fragments are able to self-assemble in well-ordered amyloid structures have supported the idea that aromatic interactions have a pivotal role in favoring, driving and orientating the amyloid fibril formation. Intermolecular stacking interactions between aromatic residues may accelerate the self-assembly process providing directionality and an energetic contribution to the aggregation process [32].

### 3.3. Amyloid Cytotoxicity

For a long while the conventional view has been that amyloid-related pathology is brought about by the intracellular and extracellular amyloid fibrils deposits. More recently, an alternative view is emerging: the main neurotoxic species would not be the insoluble aggregates themselves, but rather the soluble oligomeric species, including small globular structures, and the curvilinear structures called protofibrils.

The large body of studies on the most renowned neurodegenerative disorder, AD, has provided important contribution in identifying the amyloid species involved in toxicity and in elucidating the mechanism underlying amyloid cytotoxicity.

AD is characterized by the presence of intracellular neurofibrillary tangles (NFTs), formed by hyperphosphorylated forms of the neuronal microtubule-associated protein Tau, and of typical amyloid plaques in the extracellular space of the brain that are composed of well-ordered fibrillar aggregates of β-amyloid (Aβ) peptides.

The Aβ peptides of different lengths (39–43 residues) are produced by the intramembrane proteolysis of a transmembrane protein, amyloid precursor protein (APP), and possess a native structural disorganization. As they are natively unfolded and unstable, a conformational organization via a “partially folded” intermediate takes place during fibrillogenesis. The overproduction, accumulation and self assembly of Aβ peptides are suggested to initiate the pathogenic cascade that eventually leads to AD. According to the hypothesis of the “Amyloid Cascade”, prefibrillar diffusible Aβ assemblies cause synaptic alterations, and the concomitant plaques formation activates local inflammatory responses with the microglial and astrocytic activation. These events produce oxidative stress, disrupt neuronal homeostasis and give rise to the alteration of physiological biochemical processes leading to hyperphosphorylation and oligomerization of Tau. The aggregation of Tau reduces its binding capability to cell microtubules causing the destabilization of neuronal cytoskeleton. These synaptic and neuronal dysfunctions lead to progressive neuronal cell loss eventually ending with dementia and death [33].

In this perspective, the soluble oligomers called amyloid-derived diffusible ligands (ADDLs), protofibrils and amylospheroids are considered the main neurotoxic species responsible for the decline of memory processes of AD patients [34,35]. The neurotoxicity seems to be caused by the prefibrillar aggregates’ ability to form pores in the neuronal membrane, potentially inducing cell dysfunction via inappropriate membrane permeabilization [36]. As a matter of fact, correlations between fibrillar plaque density and severity of dementia are not always found in AD brains. On the contrary, correlations between soluble Aβ levels and the extent of synaptic loss and of cognitive impairment are stronger. It has also been reported that oligomers inhibit neuronal viability 10-fold more than fibrils and about 40-fold more than un-aggregated peptide [37].

The pivotal role of amyloid oligomers as the main culprits of cytotoxicity has been also demonstrated for several amyloid disease-related proteins, such as α-synuclein in PD, β2-microglobulin in dialysis-related amyloidosis, transthyretin in familial amyloid polyneuropathies, amylin in type II diabetes and others [38–40]. Interestingly enough, even proteins not associated with any disease are able to self-assembly and to form cytotoxic oligomers [41,42].

Increasing evidences indicate that amyloid fibrils are not inert structures inside which toxic oligomers are neutralized, but they too trigger cellular toxicity although to a lesser extent in comparison to oligomeric species. As a matter of fact, besides inducing inflammation and oxidative stress in AD, they can directly interact with cell membrane lipids and be destabilized and disassembled in the pre-fibrillar toxic forms [43–45]. Fibril disassembly can be also obtained by the action of exogenous compounds as small natural molecules and drugs [7,46]. Consequently, amyloid fibrils should be considered active sources of toxic species.

### 3.4. Therapeutic Strategies

Despite intensive efforts to face and to defeat amyloid-related diseases, and notwithstanding the remarkable advances in understanding the mechanisms underlying the pathogenesis, ultimate treatment is yet to come.

The main therapeutic strategies currently under investigation have been focused on the central events of protein misfolding and aggregation.

1. Inhibition of the amyloidogenic form of proteins by stabilization of the native conformation or by reduction in the production of misfolded/unfoldded structures.
2. Inhibition of protein self-assembly in oligomers and fibrils.
3. Enhancement in the clearance of toxic aggregates.

In this review the attention will be focused on the second approach, being aware that it is difficult to assert which is the best strategy because each of them has some limitations as well as some promising perspectives. It should moreover be noted that many efficient inhibitors of aggregation *in vitro*, often fail as therapeutic agents *in vivo*.

A synergistic combination of several approaches should be followed in order to treat efficiently amyloid diseases.

## 4. Natural Molecules

Natural aromatic-rich molecules, in particular polyphenolic compounds, constitute a large class of phytochemicals present at a high concentration in many staple foods such as such as fruit, vegetables, cereals, coffee and in a wide variety of plants. They are characterized by the presence of one or more phenolic rings which may interact with the aromatic residues of the amyloidogenic polypeptides interfering and affecting the self-assembly process. Further, several components, including electrostatic, van der Waals and solvophobic forces may promote weak, non-covalent interactions between aromatic natural molecules and proteins and perturb the hydrophobic forces that maintain in close contact the side chains of the polypeptides thus having a disrupting effect [47,48].

In what follows, some case examples among the numerous studies performed on the use of small aromatic molecules to inhibit or block or impair aggregation processes of amyloid-related proteins or peptides will be concisely reported.

### 4.1. Epigallocatechin-3-Gallate (EGCG)

**Figure 2:**
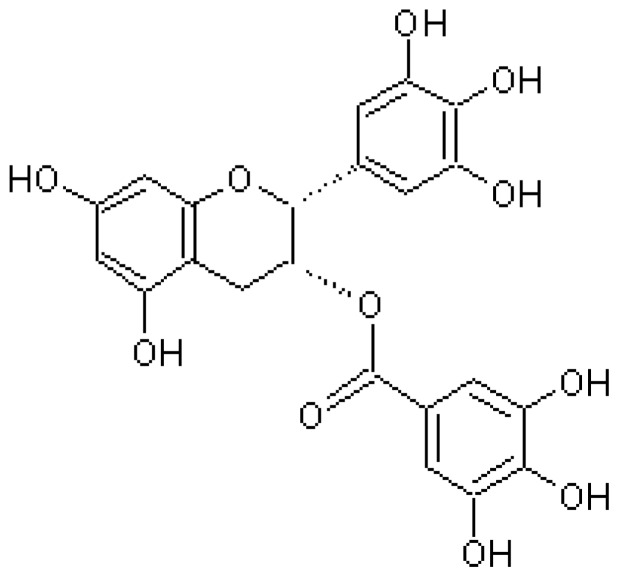
Chemical structure of EGCG.

Epigallocatechin-3-gallate (EGCG) is the most important polyphenol in green tea and shows numerous beneficial health effects and anti-aging activity through different pathways, as it is antioxidant, anti-inflammatory, and elicits amyloid protein remodeling activity (Figure 2).

EGCG has been shown to inhibit the aggregation and toxicity of multiple amyloidogenic proteins, including Aβ, α-synuclein, huntingtin, islet amyloid polypeptide, and transthyretin [49–52].

EGCG directly binds to natively unfolded Aβ, promoting the peptide self-assembly in large, spherical oligomers. These oligomers appear to be off-pathway species, unable to form fibrils and non-toxic to cells [49]. It has been shown that the administration of EGCG to transgenic mice (Tg2576) decreases Aβ levels and reduces amyloid plaques *in vivo*[53]. EGCG (Sunphenon) is presently in phase 2 clinical trials for AD.

EGCG is also able to remodel mature α-synuclein and amyloid-β fibrils and to reduce their cellular toxicity. It has been suggested that EGCG binds to β-sheet rich aggregates and promotes their structural modification, disassembling them into smaller amorphous protein aggregates that are non-toxic to mammalian cells [54].

In the case of islet amyloid polypeptide/amylin (IAPP) involved in type II diabetes, EGCG inhibits amyloid formation when added to the lag phase and it has been shown to bind to intermediates as well as to monomers and mature fibers. Interactions with aromatic residues, the disulfide, protein amino groups, or the tyrosine side chain are not required for effective inhibition by EGCG. It appears that EGCG interacts with IAPP by hydrogen bonding to the peptide backbone and by relatively nonspecific, presumably hydrophobic, interactions with side chains. It has also been found that EGCG protects cultured cells against IAPP amyloid toxicity [51,55].

### 4.2. Curcumin

**Figure 3:**
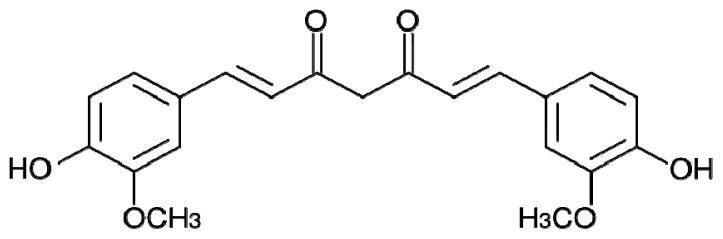
Chemical structure of curcumin.

Curcumin is a polyphenolic compound representing 75%–80% of the active components present in turmeric, used as a curry spice in food and derived from the rhizome of the plant *Curcuma longa*, a member of ginger family. Curcumin is well known for its anti-inflammatory, antioxidant, chemopreventive and chemotherapeutic properties. Its symmetric and extended conjugated molecular structure resembles the structure of Congo red, which has been commonly used in diagnosis of amyloid-related diseases for its capability to bind amyloid fibrillar structures (Figure 3).

Several studies have shown that curcumin inhibited Aβ aggregation, disaggregated fibrils and prevented Aβ oligomer formation and toxicity in a dose-dependent manner [56].

In a recent study, the effect of curcumin on α-synuclein, a compact disordered protein involved in PD, has been investigated. This protein is prone to aggregation because, during its slow reconfiguration rate, the time of the hydrophobic residues’ exposition is long enough to allow intermolecular interactions that eventually lead to fibril formation. It has been found that curcumin strongly binds to the monomer favoring intramolecular interaction and thus increasing the protein reconfiguration rate. Faster reconfiguration allows the proteins to escape from bimolecular self-assembly and prevents further aggregation steps. The result of this curcumin-induced conformational rearrangement is the inhibition of α-synuclein oligomerization and fibrils formation [57].

The conversion of the normal folded cell protein, Prion protein (PrP), into amyloid aggregates is at the basis of transmissible spongiform encephalopathies such as Creutzfeld–Jakob disease. Prion protein transition into β-sheet rich forms has been described as a complex process [58], and curcumin has been shown to be able to bind to a partially unfolded α-helical intermediate blocking its conversion to β-sheet oligomer, as well as to interact with prion fibrils preventing their further growth [59].

### 4.3. Resveratrol

**Figure 4:**
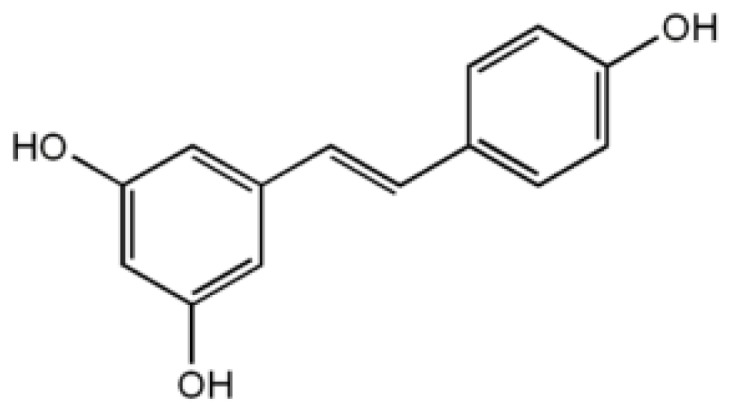
Chemical structure of resveratrol.

Resveratrol is a polyphenolic flavonoid found in grapes, red wine, mulberries, peanuts, and rhubarb. In recent years, it has been the subject of intense interest due to a range of unique anti-aging properties. These include cardiovascular benefits, phytohormonal actions, anticancer properties, antimicrobial effects and possible benefits on AD by inhibition of beta-amyloid neurotoxicity and direct effects on neural tissues (Figure 4).

Several studies have shown resveratrol to play a neuroprotective role against AD [60], as this compound could attenuate Aβ peptide-induced toxicity [61], promote Aβ clearance [62] and reduce senile plaques in cell or AD mouse models [63,64]. Recently it has also been demonstrated that resveratrol could directly bind to both monomeric and fibrillar amyloid structures [65]. In particular resveratrol seems to be able to selectively remodel soluble oligomers, fibrillar intermediates and amyloid fibrils into an unstructured, high molecular weight aggregated species that is non-toxic [66]. The study “to evaluate the impact on biomarkers of resveratrol treatment in patients with mild to moderate Alzheimer’s Disease” is in phase 2 clinical trials and is currently recruiting participants (ClinicalTrials.gov Identifier:NCT01504854).

Resveratrol seems to exert a beneficial effect also on some systemic amyloidoses. The transthyretin amyloidoses are characterized by the extracellular deposition of aggregates formed by the plasmatic homotetrameric protein transthyretin (TTR) in peripheral nerves and the heart. Resveratrol and its analogs have been found to stabilize and promote the formation of the native tetrameric TTR inhibiting their self-assembly in the cytotoxic species. As shown in the case of Aβ peptides, resveratrol may also accelerate the formation of large soluble non-toxic aggregates [67].

Furthermore, resveratrol seems to be able to interact with IAPP implicated in type II diabetes and to prevent fibril formation and cytotoxicity [68]. From simulations studies it has been hypothesized that resveratrol molecules interfere with and block IAPP intersheet side-chain stacking interaction, especially those of the aromatic rings, thus reducing the overall aggregation [69].

### 4.4. Hypericin

**Figure 5:**
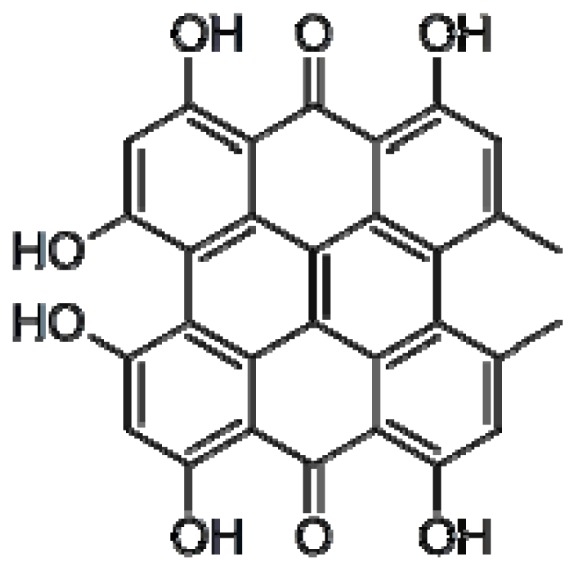
Chemical structure of hypericin.

Hypericin is a polycyclic aromatic molecule extracted from *Hypericum perforatum*, widely used as a mild antidepressant and suggested to have a neuroprotective effect [70,71] (Figure 5). It is a natural photosensitizing pigment which has been extensively studied because of its antiviral and antibacterial properties and its possible application in cancer photodynamic therapy [72].

Hypericin has fluorescent properties that make this pigment a quite reliable probe: in aqueous solutions, in fact, hypericin molecules give rise to polydispersed non-fluorescent aggregates, but they easily associate to and interact with a large variety of biomolecules by means of weak non-covalent interactions, thus yielding highly fluorescent monomers [73].

Our findings seem to indicate that this pigment can associate to early precursors of the β-sheet fibrils and/or protofibrils of Aβ peptides through stacking and hydrophobic interactions with the peptides and perturb the aggregation process thus having an inhibitory effect [74]. Hypericin does inhibit fibrillogenesis through interactions with β-sheet portions of Aβ peptide oligomers. In the aggregation process, the fraction of Aβ oligomers (dimers, trimers, and tetramers) increases and so does the β-sheet percentage and, consequently, the number of binding sites for hypericin increases [75].

### 4.5. Ferulic Acid

**Figure 6:**
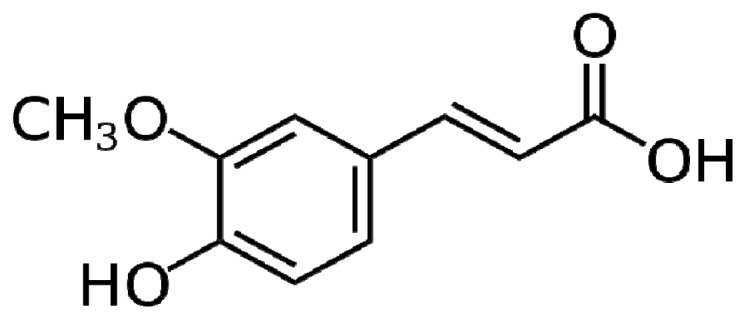
Chemical structure of Ferulic Acid (FA).

Ferulic acid (FA) is a ubiquitous phenolic plant constituent that exhibits a wide range of therapeutic effects against cancer, diabetes, cardiovascular and neurodegenerative diseases (Figure 6).

The most studied biological activities of FA are its potent antioxidant and anti-inflammatory properties that make it an interesting and promising candidate for prevention and/or treatment of disorders linked to oxidative stress, including AD [76]. It has been shown that FA protects neurons against the Aβ-induced oxidative stress and neurotoxicity *in vitro*[77] and inhibits the Aβ oligomer-induced cellular and synaptic toxicities. In fact, the long term administration of FA protects mice against Aβ-induced learning and memory deficits *in vivo*[78]. FA entrapped in solid lipid nanoparticles, used as a drug delivery system, reduces oxidative stress and cell death induced by Aβ oligomers in the neuroblastoma cell line [79].

Inhibition of formation and destabilization of amyloid fibrils by the direct binding of FA to these structures have been suggested by Ono and coworkers [80–83].

A possible explanation of FA anti-cytotoxic effects is that this phenol inhibits the formation of highly toxic oligomeric Aβ and thereby prevents lipid peroxidation, which is key to the process of triggering oxidative stress by Aβ.

Recent preliminary results of ours seem to indicate that FA redirects the organized conformation of the Aβ fibrils towards amorphous oligomers by means of hydrogen bonding and hydrophobic interactions with the various regions of the oligopeptide assembly. FA molecules cover the fibrillar structure and show a slight tendency to insert their carboxyl and hydroxyl moieties between the Aβ peptide chains. This effect could limit or even hinder the association of incoming Aβ peptides and thus inhibit fibrillogenesis, as well as their re-association in the association–dissociation dynamic equilibrium.

## 5. Conclusions

Nature may be considered an extraordinary, immense library rich in chemical compounds—many of them still unknown—that are selected and validated by evolution to match a large variety of biological functions. The intrinsic “biocompatibility” of natural molecules enable them to specifically target a wide number of biological macromolecules. In fact, natural molecules have structural and physicochemical properties, making them capable to bind to and to embed into the molecular framework of living systems. Thus, they can also interfere with physiological as well as pathological processes: “Many drugs used today are natural products or natural-product derivatives” [1].

The exploration of this natural database is not only interesting for a curiosity-driven knowledge, but can be a source of inspiration for designing and developing innovative and effective therapeutic strategies. In the case of amyloid-related diseases, several natural aromatic molecules can inhibit and delay the abnormal self-assembly of peptides and proteins into toxic oligomers and fibrillar polymers or redirect the aggregation pathway toward the formation of non-toxic protein clusters. Understanding the mechanisms underlying natural molecules’ anti-aggregation activities, as well as their anti-cytotoxic and clinical effects, optimizing their delivery in the target tissue/organ and minimizing their possible side effects, also through quality control standardization, are crucial steps for the research of future treatments against amyloidogenesis.

## Figures and Tables

**Table 1:** Main amyloid diseases and the peptides and proteins associated.

Pathology	Peptides/Proteins
Alzheimer’s Disease	Amyloid β peptide, Tau protein
Parkinson’s Disease	α-synuclein
Transmissible Spongiform Encephalopathies	Prion protein
Huntington’s Disease	Huntingtin (poliQ expansion)
Amyotrophic Lateral Sclerosis	Superoxide dismutase
Cerebellar Ataxias	Ataxins (poliQ expansion)
Type II Diabetes	Islet amyloid polypeptide/amylin
Insulin-Related Amyloidosis	Insulin
Familial Amyloid Polyneuropathies	Transthyretin
Dialysis-Related Amyloidosis	β2-Microglobulin
Primary Systemic Amyloidosis	Immunoglobulin light chain
Finnish Hereditary Systemic Amyloidosis	Gelsolin
Lysozyme Systemic Amyloidosis	Lysozyme
